# A Hybrid Recommendation System for Marine Science Observation Data Based on Content and Literature Filtering

**DOI:** 10.3390/s20226414

**Published:** 2020-11-10

**Authors:** Xiaoyang Song, Yonggang Guo, Yongguo Chang, Fei Zhang, Junfeng Tan, Jie Yang, Xiaolong Shi

**Affiliations:** State Key Laboratory of Acoustics, Institute of Acoustics, Chinese Academy of Sciences, Beijing 100190, China; songxiaoyang@mail.ioa.ac.cn (X.S.); changyongguo@mail.ioa.ac.cn (Y.C.); zhangfei@mail.ioa.ac.cn (F.Z.); tanjunfeng13@mails.ucas.ac.cn (J.T.); yangjie@mail.ioa.ac.cn (J.Y.); shixiaolong@mail.ioa.ac.cn (X.S.)

**Keywords:** recommendation system, marine science observation data, content-based filtering algorithm, spatial-temporal data

## Abstract

With the development of ocean exploration technology and the rapid growth in the amount of marine science observation data, people are faced with a great challenge to identify valuable data from the massive ocean observation data. A recommendation system is an effective method to improve retrieval capabilities to help users obtain valuable data. The two most popular recommendation algorithms are collaborative filtering algorithms and content-based filtering algorithms, which may not work well for marine science observation data given the complexity of data attributes and lack of user information. In this study, an approach was proposed based on data similarity and data correlation. Data similarity was calculated by analyzing the subject, source, spatial, and temporal attributes to obtain the recommendation list. Then, data correlation was calculated based on the literature on marine science data and ranking of the recommendation list to obtain the re-rank recommendation list. The approach was tested by simulated datasets collected from multiple marine data sharing websites, and the result suggested that the proposed method exhibits better effectiveness.

## 1. Introduction

Facing an unpredictable ocean, humans developed a variety of observation means and detection instruments to deeply explore and understand marine phenomena [1]. Remote sensing satellites, coastal ocean observatories, sea-based marine observation systems, seafloor observatories, and stereoscopic observation constitute the stereo observation systems used to perform ocean observations in different dimensions and different spatial-temporal scales [2,3,4,5,6]. For example, space-based ocean observation systems use multispectral, hyperspectral, and synthetic aperture radar (SAR) to monitor multiple marine elements of a large sea area in real-time, synchronously and continuously [7]. Coastal ocean observatories are used to realize real-time, long-term, and fixed-point monitoring [8]. Seafloor observatories perform long-term in situ monitoring of geological, physical, chemical, and biological changes in specific areas of the seabed [9]. Numerous and varying observation systems mean a large number of heterogeneous data. However, due to high data volume, users were drowning in useless data but starved of valuable data. It is challenging for researchers to identify the required data from the massive amount of ocean observation data.

Recommendation systems, which originated from the Internet and e-commerce industries, provide users with personalized information services and decision support based on data mining of magnanimity data [10]. It is an effective method to improve retrieval capabilities to help users obtain the required data, i.e., news recommendations, music recommendations, and movie recommendations [11,12,13]. With the increase of recommendation demand in various fields, scholars proposed many recommendation algorithms, such as content-based filtering (CBF), collaborative filtering (CF), and association-rule-based recommendation model (ARB) [14,15,16]. In recent years, recommendation systems have become pervasive and have been applied to spatial data [17,18]. However, recommendation systems are rarely used in marine scientific data due to their complex attributes and the lack of a large user sample. Marine scientific data have abundant attributes, including space, time, topics, and other multidimensional information. The users of marine science data mostly include educators and scientific researchers. In addition, the users’ demands for data are complex, and the demands are changing continuously with research content. In addition, marine data sharing sites often lack an evaluation system, thus it is difficult to obtain users’ behavioral preferences and sufficient user ratings. Therefore, it is difficult for traditional recommendation algorithms to solve them.

In this study, we developed a hybrid recommendation system for marine scientific data based on the CBF and CF with literature. CBF uses the information of data that has been selected by the user. CBF calculates the similarity of the data based on the attributes, then sorts the data and recommends data. CF filters and evaluates data for the user based on the interest level of other users with the same interest in data. CF calculates the similarity matrix among users based on the relationship of the existing data, finds users with common interests and hobbies, and generates recommendations that the users may be interested in [19]. Given the lack of a large user sample, a literature analysis is introduced instead of a large user sample. In this method, data similarity was calculated by analyzing the subject, source, spatial, and temporal attributes, which was used to build the initial data similarity matrix. Then data correlation, which presents the implicit relationship between marine data, was obtained by analyzing literature on marine data. The data correlation was used to optimize the data similarity matrix and build the data recommendation matrix. Finally, based on the data recommendation matrix and user’s historical data, the recommendation system generates a recommendation list for the user. This recommendation system improved the recommendation accuracy and ensured the diversity of recommended data by combing various data attributes and literature analysis.

The rest of this paper is organized as follows. In Section 2, we review the related work on the recommender algorithm and the hybrid recommender systems. The detailed description of our method is presented in Section 3. Section 4 gives experiment results, Section 5 provides a brief discussion, and Section 6 concludes this paper.

## 2. Related Work

In this section, we first introduce some related work on recommendation algorithms, and then some hybrid recommendation systems are reviewed. Additionally, we also discuss some recommendation evaluation methods.

### 2.1. Recommendation Systems

The recommendation algorithm is the foundation and core of the recommendation system—it includes CBF, CF, ARB, etc. CBF and CF are two popular methods used by personalized recommendation systems.

CBF makes recommendations by analyzing the features of the items and establishing the similarity between items. In recent years, an increasing number of studies on CBF have been performed. Yu Li et al. proposed a recommendation system of good images based on image feature combinations and achieved high accuracy in recommending similar good images at high speed [14]. Jiangbo Shu et al. used CBF to target the learning resources to the right students combined with a convolutional neural network, which was used to predict the latent factors from the text information of the multimedia resources [20]. Son Jieun et al. used a multi-attribute network to effectively reflect several attributes in CBF to recommend items to users with high accuracy and good robustness [21]. CBF is simple and easy to understand, and the new items can be recommended as well as old ones. However, it only recommends similar data, thus it has limitations on recommendation diversity. In addition, it is difficult to explore users’ potential interests.

CF is the most widely used algorithm. It only relies on interaction information between users and items, such as clicks, browsing, and rating, rather than information about users or items. CF is widely used in many fields. For example, Zhihua Hu et al. embedded CF for online clothing and verified the effectiveness of this method using a test dataset from e-commerce platforms (www.taobao.com) in China [22]. Choi Il Young et al. generated online footwear commercial video recommendations based on changes in user’s facial expression captured every moment based on CF [23]. Bach Ngo Xuan et al. proposed an efficient collaborative filtering method that exploits co-commenting patterns of users to generate recommendations for news services, and this method exhibited high accuracy [24]. CF can effectively discover the potential interests of users and recommend new items. However, CF has a cold start problem. The lack of basic information of new users would lead to low-quality recommendations, and a limited number of users make it difficult to recommend new data.

### 2.2. Hybrid Recommendation Systems

These recommendation algorithms have their own unique advantages and limitations. The hybrid recommendation systems were proposed to optimize the algorithms and address the limitations by combining two or more recommendation algorithms or introducing other algorithms. According to different targets, several hybridization methods are used to combine a variety of algorithms, such as weighted, switching, mixed, feature combination, cascade, feature augmentation, and meta-level [25]. The weighted hybrid recommendation systems calculate recommendation algorithms and weighted their results. The switching hybrid recommendation systems choose the appropriate algorithms to make a recommendation based on certain pre-defined criteria, the mixed hybrid recommendation systems will present all commendation results of mixed\algorithms, the feature combination hybrid make commendations with features from different recommendation algorithms, the cascade hybrid using one recommendation algorithm to refine another recommendation result, the feature augmentation recommendation takes one recommendation result as an input feature to another, and the meta-level recommendation system integrates one recommendation algorithm to another algorithm. The most common hybrid recommendation system is to combine CF and CBF. Bozanta Aysun et al. integrated CBF and CF together with contextual information in order to get rid of the disadvantages of each approach, and the experimental evaluation showed improvement [26]. Geetha G et al. provided more precise recommendations concerning movies to a new user and other existing users by hybrid filtering that provided a combination of the results of CBF and CF [27].

Hybrid recommendation systems can optimize recommendation results by effectively making use of additional data information and other technologies. Fang Q et al. proposed a spatial-temporal context-aware personalized location recommendation system based on personal interest, local preference, and spatial-temporal context influence [28]. As one of the advancing researches of the world, the neural network has been applied in many fields [29,30,31,32], many scholars also introduced neural network into the recommendation system. Yang Han et al. using the convolutional neural network algorithm to predict the cause and time of recurrence of cancer patients, designed an intelligent recommendation system of a cancer rehabilitation scheme [33]. Libo Zhang et al. proposed a model combining a collaborative filtering recommendation algorithm with deep learning technology to further improve the quality of the recommendation results [34] T.H. Roh et al. proposed a three-step CF recommendation model combing with self-organizing map (SOM) and case-based reasoning (CBR), and demonstrates the utility of this model through an open dataset of user preference [35].

Many of the researchers have made much effort on hybrid recommendation systems and proposed many recommendation methods, but no previous systems fit all data recommendation scenarios.

### 2.3. Recommendation Evaluation Metrics

Effective evaluation of the recommendation system is critical. Depending on the recommendation task, the most commonly used recommendation quality measures can be divided into three categories: (a) Quality of the predictions, such as mean absolute error and mean squared error. (b) Quality of recommendations, such as precision, recall, average reciprocal hit rank (ARHR). (c) Quality of the list of recommendations, such as half-life utility (HL), mean reciprocal rank (MRR), mean average precision (MAP). (d) Other assessment indicators, such as intra list similarity (ILS), mean absolute shift (MAS) [36].

Although the importance of freshness and unpredictability was proposed, there has been no systematic quantitative study of them. In this study, ARHR and ILS were adopted. ARHR is a very popular indicator to evaluate recommendation systems. It measures how strongly an item is recommended [37,38]. ILS measures the diversity of recommendation lists. If the items in the recommendation list are less similar and ILS is smaller, the more diverse the recommendation results will be [39].

## 3. Methods

The proposed method was mainly divided into 2 steps: (1) Calculate the similarity between items; (2) Generate recommendation lists for users according to the similarity of items and users’ historical behaviors. For the marine dataset, the user’s interest in a dataset was calculated by the following formula:

(1) $$R_{y}=\Sigma_{x\in D}P(x,y)U_{x},$$

where, $R_{y}$ is the user’s interest in y dataset. D is a collection of the historical datasets that the user has used, and x. P(x,y) is the value of the xth row and the yth column in the recommendation matrix. $U_{x}$ is the user’s interest in x dataset. In practice, $U_{x}$ can be set according to the user’s ratings or browsing time.

The proposed method was primarily used to calculate the recommendation matrix, which was the hardest and the most critical part of the recommendation system. The proposed method included the following 3 steps. First, the data similarity was calculated by analyzing the attributes of marine science data. Then, the data correlations between different marine science data were obtained by retrieving relevant literature from each category of marine science data. Finally, the data similarities and correlations were integrated to obtain the recommended matrix.

### 3.1. Data Similarity

Data similarity was defined based on 4 aspects of similarity, that was the subject similarity, source similarity, spatial similarity, and temporal similarity. Based on the 4 similarities, the weighted sum calculation model was used to calculate data similarity (Sim(x,y)) as Equation (2).

(2) $$Sim\left(x,y\right)=W_{1}Sim_{sub}\left(x,y\right)+W_{2}Sim_{sou}\left(x,y\right)+W_{3}Sim_{spa}\left(x,y\right)+W_{4}Sim_{tem}\left(x,y\right),$$

Here, x and y are 2 datasets, $Sim_{sub}\left(x,y\right)$ is the subject similarity of the 2 datasets, $Sim_{sou}\left(x,y\right)$ is the source similarity, $Sim_{spa}\left(x,y\right)$ is the spatial similarity, and $Sim_{tem}\left(x,y\right)$ is the temporal similarity.

In the study, $W_{1}$, $W_{2}$, $W_{3},$ and $W_{4}$ were determined subjectively by expert scoring. Through consulting several experts in different fields, $W_{1}$, $W_{2}$, $W_{3}$, and $W_{4}$ were set as 0.2, 0.2, 0.3, and 0.3, respectively.

#### 3.1.1. Subject Similarity

**Figure 1:**
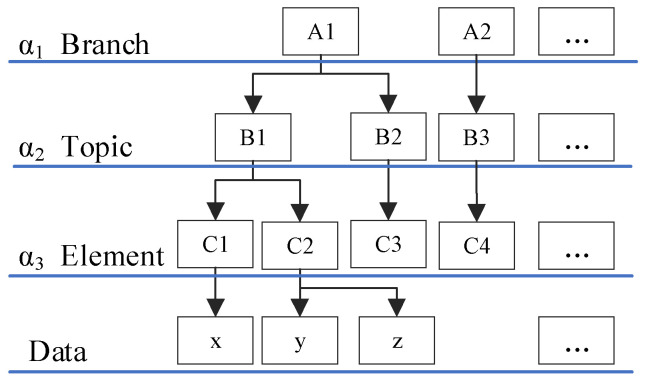
Data category hierarchy tree.

Subject similarity refers to the similarity of data category. Generally, there are 4 basic branches of marine science: Marine physics, marine chemistry, marine geology, and marine biology [40]. These branches have different research topics, such as marine meteorology, marine acoustics, marine electromagnetism, and marine optics, and different research topics contain different observation elements. The impact of the category was described by subject similarity. A category hierarchy tree was generated, and a parameter was assigned to each layer to represent the increasing hierarchy. As shown in Figure 1, the category hierarchy tree was a 4-layer structure consisting of the branch, topic, element, and data. Here, $\alpha_{1}$, $\alpha_{2}$ and $\alpha_{3}$ represented the parameters of each layer, separately. Because these parameters do not necessarily increase linearly as the depth of the category hierarchy tree increases in practical applications, $\alpha_{1}$, $\alpha_{2}$ and $\alpha_{3}$ were different [41].

According to the category hierarchy tree, the subject similarity was calculated based on the parameters from the first layer to the common layer of 2 marine data, as shown in Formula (3).

(3) $$Sim_{sub}\left(x,y\right)=\frac{c_{xy}}{\alpha_{1}+\alpha_{2}+\alpha_{3}},$$

Here, $c_{xy}$ is the sum of the parameters from the first layer to the common layer of 2 marine data. Using Figure 1 as an example, the common layer of data x and y is the second layer, which represents a topic layer. Thus, $c_{xy}$ is the sum of the parameters of the 1st layer and the 2nd layer, namely, $c_{xy}=\alpha_{1}+\alpha_{2}$.

#### 3.1.2. Source Similarity

**Figure 2:**
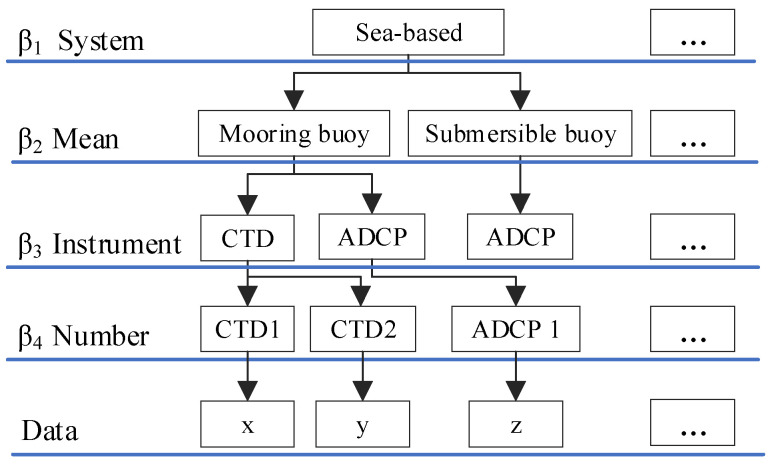
Data source hierarchy tree.

With the development of ocean technology, the increasing diversity of oceanographic instruments has led to a greater diversity of data sources with different spatial and temporal dimension properties. Thus, the similarity of data sources was crucial. The source hierarchy tree can be divided into 5 layers: (a) Observation system, including space-based systems, shore-based systems, air-based systems, sea-based systems, ship-based systems, seabed-based systems, and reanalysis systems, with new data obtained by multidata fusion and analysis; (b) observation means, including mooring buoys, submariners buoys, voyage, stations, and numerical simulation; (c) instrument mounted on observation means, such as acoustic doppler current profiler (ADCP), conductivity-temperature-depth system (CTD), and ocean color imager; (d) specific instrument number; (e) Datasets. For example, the space-based observation method included the Seastar satellite, OceanSat series satellites, and HY-1 satellites. Sea- and seabed-based observation methods included several CTDs, ADCPs, and other different in situ observation instruments [42]. The source hierarchy tree was constructed, as shown in Figure 2.

According to the source hierarchy tree, the source similarity was calculated based on parameters for each marine dataset, as shown in Formula (4).

(4) $$Sim_{sou}\left(x,y\right)=\frac{s_{xy}}{\sum_{i=1}^{4}\beta_{i}},$$

Here, $\beta_{i}$ is the parameter of layer i, and $s_{xy}$ is the sum of the parameters from the 1st layer to the common layer of 2 marine datasets. Using Figure 2 as an example, $s_{xy}$ is the sum of $\beta_{1}$, $\beta_{2}$,and $\beta_{3}$.

#### 3.1.3. Spatial Similarity

To calculate spatial similarity, the compatibility of the spatial scale and the overlap of the spatial range were analyzed. Based on the geometric type, marine data can be divided into a point, line, polygon, and three-dimensional(3D) volume, which was the most striking feature of marine scientific data. When calculating the spatial similarity, the compatibility of the space scale was the primary consideration factor. The spatial similarity of the 2 datasets were unidirectional. For instance, data x and y were 2 marine scientific datasets, data x is line data, and data y is point data. For data x, the similarity to data y is 0. For data y, the similarity to data x is calculated by the distance between these datasets.

(1) Data x is point data:

The spatial similarity was calculated by analyzing the distance between two data points. Argo floats data were the most commonly used point marine data, and the Argo floats were fixed by satellites when they were drifting on the surface with errors of 150 m to 1000 m [43]. Thus, in this study, the similarity was considered as 0 if the distance between 2 data points was greater than 1 km. Thus, the spatial similarity was calculated using the following formula:

(5) $$Sim_{spa}\left(x,y\right)=\{\begin{array}{c} \;0\;\;\;\;\;\;\;\;\;\;\;,\;\;D_{xy}\geq 1\\ 1-D_{xy},\;\;D_{xy}<1 \end{array},$$

where $D_{xy}$ is the distance of data x and y, and the unit is km.

(2) Data x is line data:

For line data, the similarity was calculated by the overlap length as shown in Formula (6). If data y is point data, the similarity is 0.

(6) $$Sim_{spa}\left(x,y\right)=\frac{L\left(x\cap^{\text{​}}y\right)}{L_{x}},$$

Here, $L\left(x\cap^{\text{​}}y\right)$ is the overlap length of data x and y, and the unit is km.

(3) Data x is polygon data:

For polygon data, the similarity was calculated based on the overlap area (Formula (7)). Considering the compatibility of space scale, data y must be polygon data or 3D volume. Otherwise, the similarity is 0.

(7) $$Sim_{spa}\left(x,y\right)=\frac{A\left(x\cap^{\text{​}}y\right)}{A_{x}},$$

Here, $A\left(x\cap^{\text{​}}y\right)$ is the overlap area of data x and y. $A_{x}$ is the area of data x.

(4) Data x is 3D volume data:

To compute spatial similarity of 3D volume data, it is essential to ensure that data y is also 3D volume data; otherwise, the similarity is 0. The similarity is calculated based on the overlap volume using Formula (8).

(8) $$Sim_{spa}\left(x,y\right)=\frac{V\left(x\cap^{\text{​}}y\right)}{V_{x}},$$

Here, $V\left(x\cap^{\text{​}}y\right)$ is the overlap volume of data x and y. $V_{x}$ is the volume of data x.

#### 3.1.4. Temporal Similarity

Similar to the calculation of spatial similarity, the calculation of temporal similarity also needs to consider scale and range. The scale of the data generally includes seconds, minutes, hours, days, decades, months, and years, corresponding to levels 1~7. Before the temporal similarity calculation, it is necessary to judge whether the time scales are the same or compatible. For example, the annual data can be calculated by monthly data, but monthly data cannot be obtained from annual data, thus the similarity of annual data for monthly data is 0. The temporal similarity can be expressed, as noted in Formula (9).

(9) $$Sim_{Tim}\left(x,y\right)=\{\begin{array}{c} \;\;\;0\;\;\;\;\;\;\;\;\;\;\;\;\;\;\;\;\;\;\;\;\;\;\;\;\;\;\;\;\;\;\;\;,\;\;TS_{x}<TS_{y}\\ \frac{7-\left(TS_{x}-TS_{y}\right)}{7}\times \frac{\left|T_{x}\cap^{\text{​}}T_{y}\right|}{\left|T_{x}\right|},\;\;TS_{x}\geq TS_{y} \end{array},$$

### 3.2. Data Correlation

To ensure the diversity of the recommendation data, data correlation was added to the proposed method. In interdisciplinary studies, data with less content similarities were often used together. In this study, the published papers were used to reflect the use of marine science data comprehensively. A large number of relevant studies were retrieved using the authoritative literature database, and data were applied and correlated based on statistical analysis. A co-occurrence matrix was used to describe the correlation between data, and data correlation was assessed using Formula (10) [44,45].

(10) $$Ass\left(x,y\right)=\frac{N\left(L_{x}\cap^{\text{​}}L_{y}\right)}{\sqrt{N_{x}N_{y}}},$$

Here, $N_{x}$ and $N_{y}$ are the number of literatures for data x and y, respectively. $L_{x}$ and $L_{y}$ are literature of data x and y, respectively. $\;N\left(L_{x}\cap^{\text{​}}L_{y}\right)$ is the number of the same literature of data x and y.

### 3.3. Data Recommendation Model

In this study, the data recommendation matrix is a result of the joint action of data similarity and data correlation and was calculated by the weighted average method. The weights of data similarity and data correlation were set by their contribution. The number of relevant studies will affect the weight of data correlation in data recommendation. A limited number of studies will lead to low reliability of data association. In this case, the data similarity played an important role in the data recommendation model. Thus, the weight of data correlation varies with the number of relevant literature. In this case, it was assumed that the data similarity and data correlation have the same contribution to the data recommendation model. The data recommendation matrix is constructed as follows:

(11) P(x,y)=0.5×Sim(x,y)+0.5×Ass(x,y),

where, Sim(x,y) is the data similarity of data y for data x, Ass(x,y) is the data correlation between data x and data y. P(x,y) is used to build a recommendation matrix, and the data recommendation matrix can be represented by formula (12).

(12) $$P=\left(\begin{array}{cc} \begin{array}{cc} P\left(D_{1},D_{1}\right) & P\left(D_{1},D_{2}\right)\\ P\left(D_{2},D_{1}\right) & P\left(D_{2},D_{2}\right) \end{array} & \begin{array}{cc} \cdots & P\left(D_{1},D_{n}\right)\\ \cdots & P\left(D_{2},D_{n}\right) \end{array}\\ \begin{array}{cc} \vdots & \vdots \\ P\left(D_{n},D_{1}\right) & P\left(D_{n},D_{2}\right) \end{array} & \begin{array}{cc} \ddots & \vdots \\ \cdots & P\left(D_{n},D_{n}\right) \end{array} \end{array}\right),$$

Here, $D_{1}\sim D_{n}$ are all datasets.

**Figure 3:**
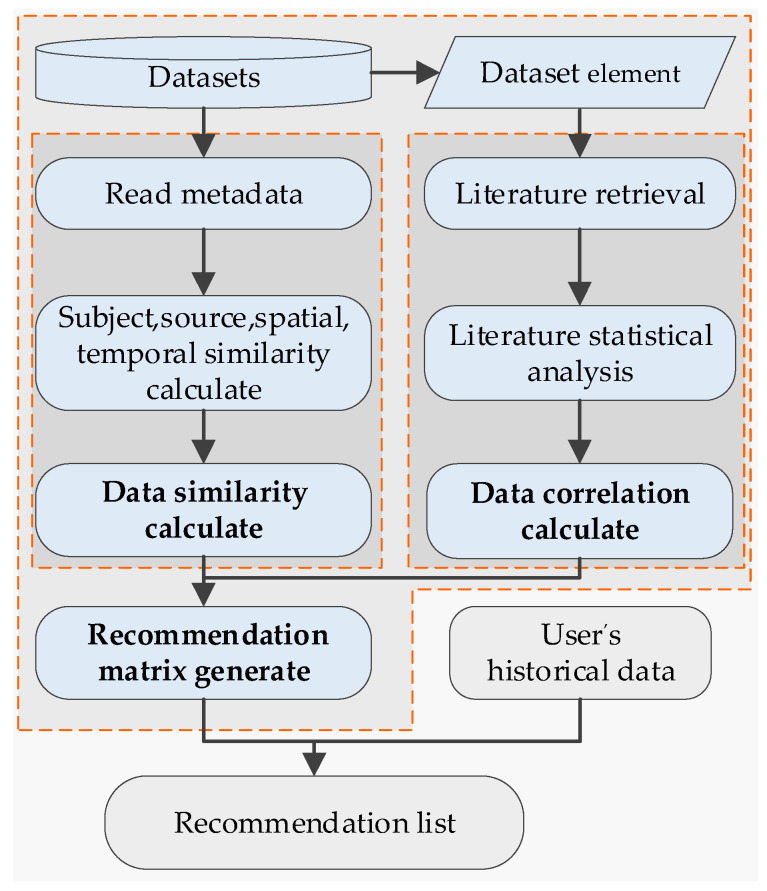
Recommendation process.

Based on the data recommendation matrix and user’s historical data, the recommendation system generates a recommendation list for the user, as shown in Figure 3.

### 3.4. Evaluation Metrics

Referring to the calculation method of average reciprocal hit rank (ARHR), the distribution of the same element (DE) was used to evaluate the distribution of the datasets, which belonged to the same element. DE was used to describe the sensitivity of the data sequence. The formula is noted as follows:

(13) $$D_{i}=\sum_{j\in L}\frac{L_{j}}{P_{j}}$$

where for dataset i, L is the ith row in recommendation matrix. $P_{j}$ is the position of the dataset j within list *L*, $L_{j}$ is the jth dataset in L, $L_{j}$ belongs to the same element as dataset i.

ILS was used to reveal the diversity of the recommended list. A smaller ILS indicates increased diversity of recommended results. The formula is noted as follows:

(14) $$M_{i}=\frac{\sum_{x\in L,y\in L,x\neq y}Sim\left(x,y\right)}{\sum_{x\in L,y\in L,x\neq y}1}$$

where, Sim(x,y) is the data similarity of data y for data x, L is the ith row in recommendation matrix. Obviously, the lower values of DE and ILS indicate better performance of the model.

## 4. Experiments

### 4.1. Datasets

To verify the effectiveness of the proposed recommendation algorithm, 20 simulated datasets were collected from multiple marine data sharing websites, including the National Marine Data Center [46], China Argo Real-time Data Center [47], and the seafloor observation network experiment system [48]. The datasets commonly used observational elements, including submarine topography data, temperature observation data, and salinity observation data, as shown in Table 1.

Originally, the dataset has different contents with a different format. To expand and normalize the content, the attributes of 20 datasets were refactored into the properties file, including subject, source, spatial scale, spatial range, depth, temporal scale, and temporal range. A sample of the generated dataset is shown in Table 2.

### 4.2. Results

#### 4.2.1. Recommendation Based on Data Similarity

(1) Subject similarity results

**Figure 4:**
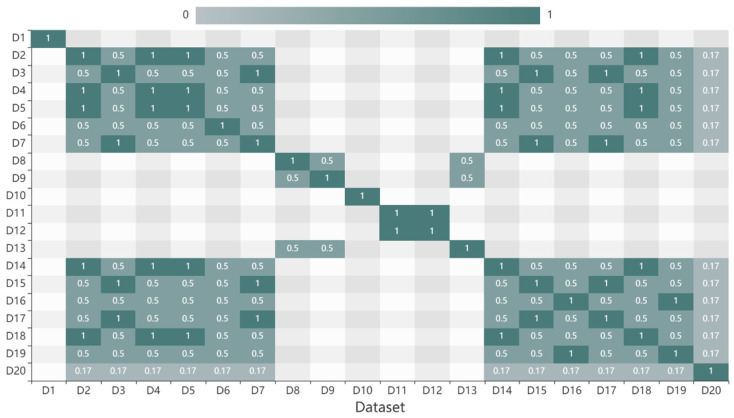
Subject similarities of datasets.

According to the subject classification of observed elements, the category hierarchy tree was built, and the weights of the three layers were set as 1, 2, and 3. The datasets were classified according to the category hierarchy tree. Then, the subject similarity was calculated, and the result was presented in Figure 4. The subject similarities of two datapoints are reciprocal. D2, D4, D5, D14, and D18 are different temperature datasets located on the third layer of the hierarchical, thus the subject similarity between two of them is 1. D2 and D3 are different elements that belong to the same topic “Physical Property” of branch “Physical Oceanography”, thus the subject similarity between them is 0.5. D20 belongs to the topic “Marine Meteorology” of the branch “Physical Oceanography”, thus the subject similarity between D2 and D20 is 0.17. When two data belong to different branches, the subject similarity is 0, such as D10 and other data.

(2) Source similarity results

**Figure 5:**
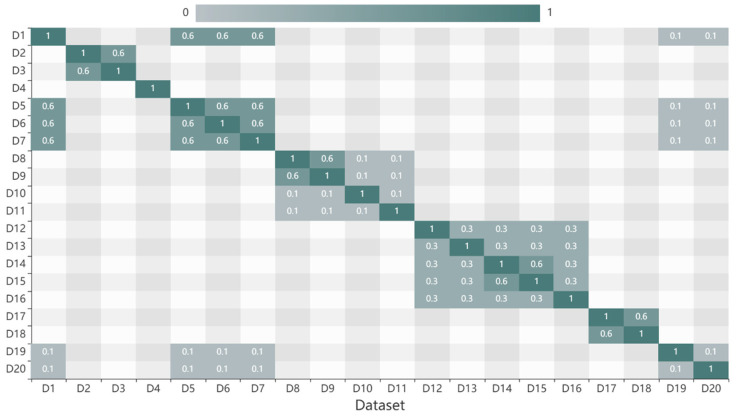
Source similarities of datasets.

Dataset source has a significant influence on the data similarity; thus, it is necessary to build the source hierarchy tree and calculate the source similarity. Similar to the category hierarchy tree, the weights of the four layers of the source hierarchy tree were set as 1, 2, 3, and 4. The results shown in Figure 5 indicate that the distribution of spatial similarities is symmetric. Because the datasets were composed of data from three shared websites, and the data sources were diverse, the source similarities between datasets were generally minimal. Several data based on the same observation means exhibited high source similarity with each other. D12, D13, D14, D15, and D16 were obtained from different instruments of the seafloor observation network experiment system, thus the source similarities were 0.3 or 0.6.

(3) Spatial similarity results

**Figure 6:**
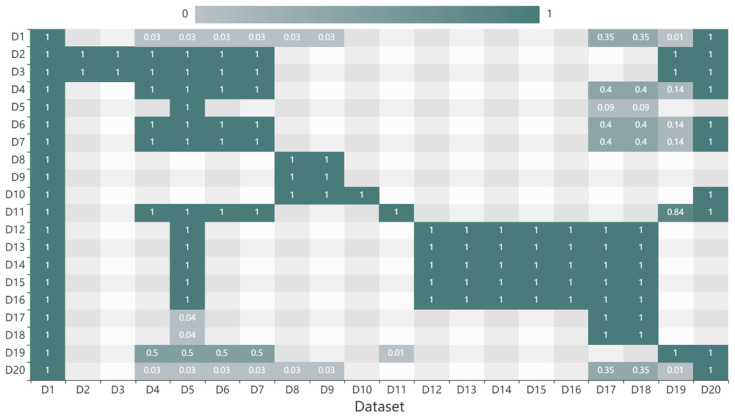
Spatial similarities of datasets.

Because the spatial similarity of two data was unidirectional, the distribution of spatial similarities was asymmetric, as shown in Figure 6. D1 is the global digital elevation model (DEM) data with a resolution of 30° and provides the foundation for other data. Thus, there is no need to consider the depth and spatial scale. For all these reasons, the similarities between D1 and other data are 1. D12, D13, D14, D15, and D16 are point data, and the locations of the instruments are close. Thus, the spatial similarities of these are greater than 0.9. Here, D5 is polygon temperature data from the northwest Pacific (longitude 99°–150°N and latitude 10°–52°E), and D12 is point data (located at 111.07°N and 17.58°E). Thus, for D12, the spatial similarities with D5 are 1. However, for D5, the spatial similarities with D12 are 0. The depth of D2 and D12 are different, and their spatial similarities are 0.

(4) Temporal similarity results

**Figure 7:**
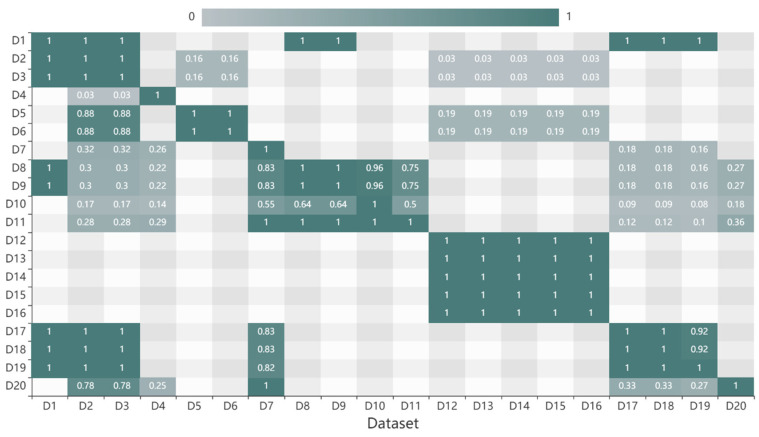
Temporal similarities of datasets.

Temporal similarities of the dataset were calculated based on the length of overlap time. Figure 7 illustrates source similarity results. As the basic data, D1 has no time range attribute. D1 is global DEM data in 2014. The temporal similarity depends on whether the time of D1 is within the time range of other data, thus the temporal similarities between D1 and other data are 0 or 1.

(5) Dataset recommended list

**Figure 8:**
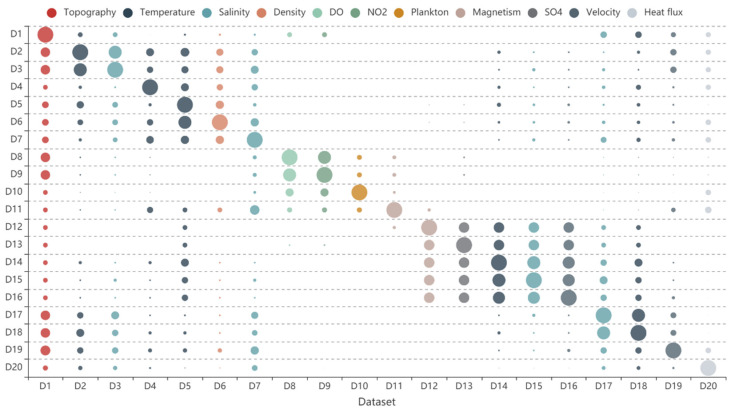
Data similarities of datasets.

Data similarity is defined using the weight sum method based on subject similarity, source similarity, spatial similarity, and temporal similarity. The result is shown in Figure 8. Each row represents the similarity of each dataset for the dataset, e.g., row 1 represents the similarity of each dataset (D1–D20) for D1. In addition, each column is colored to indicate the different elements to which the dataset belongs (Table 1). Figure 8 illustrated that most of the data similarities are within the range of 0.1 to 0.6. The similarities of D8, D9, D10, and D11 are 0 for most of the other data. These data are marine chemical environmental data with differences in subject, source, spatial and temporal aspects between them and other data. D12, D13, D14, D15, and D16 still exhibit high similarities between each other. Based on the data similarity, the recommended list was obtained.

#### 4.2.2. Re-Ranked Dataset Recommended List

The dataset recommended list was re-ranked by data correlation based on the use of data in the literature. Based on the authoritative literature database of China National Knowledge Network (CNKI), this paper performed statistical analyses on the use of Marine science data and comprehensively reflected the use of marine science data. The type of retrieval strategy was an element (Table 1), and the research field was set as oceanography. A co-occurrence probability matrix was used to represent the correlation between elements, as shown in Table 3.

**Figure 9:**
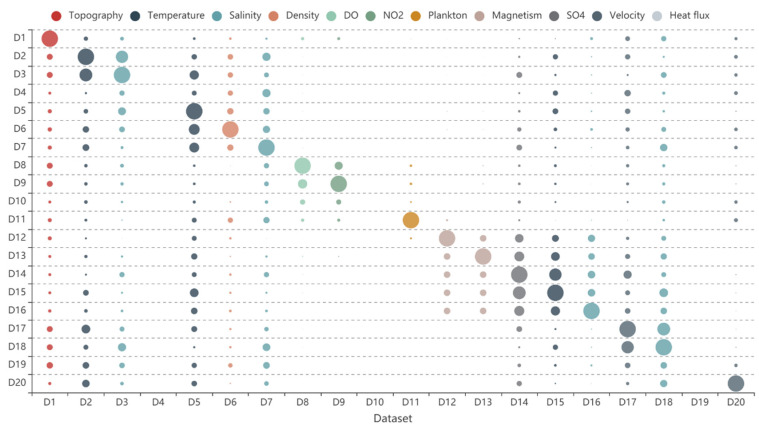
Data similarities of datasets.

Then, the co-occurrence probability matrix was introduced into the data recommendation model to obtain the final data recommendation result. As noted in Figure 9, most of the similarities were small and ranged from 0.1 to 0.6. Only a few similarities were in the range of 0.7 to 0.9, such as similarities between D2 and D3, D14 and D15, and D18 and D19.

## 5. Discussion

Based on the data similarity and data correlation, the data recommendation results before and after adding data correlations were obtained. The elements of temperature and salinity included multiple datasets, thus elements E1 and E2 were used as examples. In the recommended list for D2, which is a temperature dataset, the top 10 recommendation results included D4, D5, D14, and D18 temperature datasets. However, in the re-ranked recommended list for D2, only D4 and D5 were included in the list of top 10 results. Instead of D14 and D18, D15 and D17 salinity datasets were included in the top 10. The recommended list for other datasets that belonged to the same element exhibited the same sorted results.

**Figure 10:**
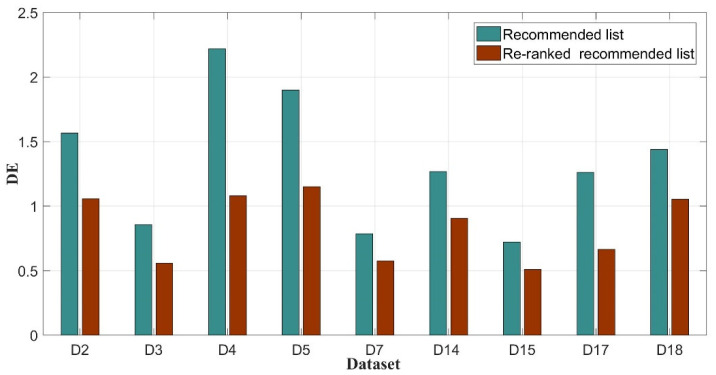
DE of two recommended lists.

To describe this distribution and diversity of recommended results, DE and ILS were used to evaluate the recommended results. Figure 10 presents the DE results of several datasets in the recommended list and re-ranked the recommended list. DEs of the dataset in the recommended list are increased compared with those in the re-ranked recommended list. DEs of D4, D5, and D17 in two recommended list have obvious differences. DE of D4 in the recommended list is 2.22. In contrast, D4 in the re-ranked recommended list has a DE of 1.08. For D5, DE in the recommended list is 1.9, whereas DE in the re-ranked recommended list is 1.15. DE of D17 in the recommended list is 1.26, and DE of D17 in the re-ranked recommended list is 0.67. Although DEs of other datasets exhibit relatively small differences, the smallest difference was 0.21, which was noted as the DE difference of D7 and D15. The average DE of several datasets in the recommended list is 1.34, and the average DE in the re-ranked recommended list is 0.84. In short, the distribution of datasets in the re-ranked recommended list is superior to that in the recommended list.

**Figure 11:**
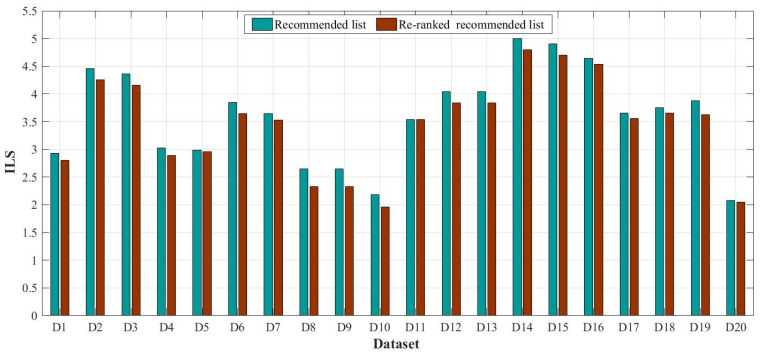
ILS of two recommended lists.

In this study, the top 10 datasets in the two recommended lists are considered as valid recommendation datasets. Thus, based on the top 10 datasets, ILS was calculated, and the result is shown in Figure 11 below. ILSs of all datasets in the recommended list is greater than ILSs in the re-ranked recommended list. ILSs with the greatest differences include ILSs of D8 and D9, and the value is 0.32. ILSs of D5, D11, and D20 in the two recommended lists exhibit minimal differences, and the value is approximately 0.01. On the whole, the average of ILSs of all datasets in the recommended list is 3.61, whereas the average in the re-ranked recommended list is 3.45.

## 6. Conclusions

In this study, the recommendation system is proposed for marine science observation data based on data similarity and data correlation. According to our method, the dataset recommendation list was obtained using data similarity. Then, the dataset recommendation list was ranked by data correlation to obtain a re-ranked dataset recommended list. We conducted experiments on 20 simulated datasets, and DE and ILS were used to evaluate the effectiveness. The result shows that the average DE of several datasets belonging to two elements in the recommended list is 1.34, and the average DE in the re-ranked recommended list is 0.84. The distribution of datasets in the re-ranked recommended list is superior to that in the recommended list. The average of ILSs of all datasets in the recommended list is 3.61, whereas the average in the re-ranked recommended list is 3.45. The diversity of the recommended list is greater than that of the re-ranked recommended list. In summary, the proposed method exhibits better effectiveness.

The recommendation system we proposed combined the data attributes and data applications, and we tested the recommendation system with simulated datasets from three marine data sharing websites. Therefore, a next step would be to expand the experiments with more comprehensive data from several other marine data sharing websites, such as the national oceanic and atmospheric administration (NOAA), Ocean Networks Canada (ONC), and Monterey Bay Aquarium Research Institute (MBARI). In addition, this method can be applied to a wide range of science data scenarios similar to marine data, and it is well-suited to the data sharing websites that lack users or lack a user rating system.

## Figures and Tables

**Table 1:** Introduction of datasets.

ID	Element	Datasets
E0	Submarine topography data	D1
E1	Temperature	D2, D4, D5, D14, D18
E2	Salinity	D3, D7, D15, D17
E3	Density	D6
E4	Dissolved oxygen (DO)	D8
E5	NO_2_ concentration	D9
E6	Plankton	D10
E7	Magnetism	D11, D12
E8	SO_4_ concentration	D13
E9	Velocity	D16, D19
E10	Heat flux	D20

**Table 2:** Samples of datasets.

ID	Element	Instrument Number	Spatial Scale	Longitude	Latitude	Depth	Temporal Scale	Time Range
D1	E0	5000	1	−180~180	−90~90	-	-	2014
D2	E1	3000	0	120.4	36	0	1	1996.01.01~2019.10.31
D3	E2	3000	0	120.4	36	0	1	1996.01.01~2019.10.31
D4	E1	0000	1	99~150	10~52	0	2	1982.01.06~1996.6.26
D5	E1	5001	2	99~150	10~52	0~9000	1	2016.01.08~2020.05.08
D6	E3	5002	1	99~150	10~52	0	1	2016.01.08~2020.05.08

**Table 3:** Cooccurrence probability matrix.

Element	E0	E1	E2	E3	E4	E5	E6	E7	E8	E9	E10
E0	1.00	0.20	0.13	0.19	0.01	0.00	0.00	0.05	0.00	0.44	0.00
E1	0.01	1.00	0.65	0.19	0.07	0.00	0.00	0.00	0.00	0.00	0.08
E2	0.00	0.72	1.00	0.16	0.08	0.00	0.00	0.00	0.00	0.00	0.02
E3	0.01	0.42	0.32	1.00	0.04	0.00	0.00	0.01	0.00	0.17	0.01
E4	0.00	0.38	0.43	0.11	1.00	0.01	0.00	0.00	0.00	0.06	0.00
E5	0.00	0.40	0.36	0.00	0.20	1.00	0.00	0.00	0.00	0.04	0.00
E6	0.00	0.48	0.28	0.20	0.04	0.00	1.00	0.00	0.00	0.00	0.00
E7	0.14	0.31	0.05	0.36	0.02	0.00	0.00	1.00	0.00	0.12	0.00
E8	0.00	0.50	0.33	0.17	0.00	0.00	0.00	0.00	1.00	0.00	0.00
E9	0.05	0.39	0.26	0.26	0.03	0.00	0.00	0.00	0.00	1.00	0.01
E10	0.00	0.75	0.15	0.08	0.01	0.00	0.00	0.00	0.00	0.02	1.00

## References

[1] Venkatesan R., Tandon A., Sengupta D., Navaneeth K.N. (2018). Recent Trends in Ocean Observations.

[2] Qi S.-P., Li Y.-Z. (2019). A review of the development and current situation of marine environment observation technology and instruments. Shandong Sci..

[3] Schofield O., Glenn S. (2004). Introduction to special section: Coastal ocean observatories. J. Geophys. Res..

[4] Pan D. (2003). Satellite marine remote sensing in China. Proc. SPIE Int. Soc. Opt. Eng..

[5] O’Driscoll R.L., Peter D.J., Richard N., Macaulay G.J., Dunford A.J., Marriott P.M., Craig S., Miller B.S. (2012). Species identification in seamount fish aggregations using moored underwater video. Ices J. Mar. Sci..

[6] Monna S., Falcone G., Beranzoli L., Chierici F., Cianchini G., De Caro M., De Santis A., Embriaco D., Frugoni F., Marinaro G. (2014). Underwater geophysical monitoring for European Multidisciplinary Seafloor and water column Observatories. J. Mar. Syst..

[7] Xiao C., Sun D., Wang S., Qiu Z., Yu H., Zhang J. (2018). Long-term changes in colored dissolved organic matter from satellite observations in the Bohai Sea and North Yellow Sea. Remote Sens..

[8] Yusri Y., Stephen K.J., Alkarkhi A.F.M. (2018). Experimental data on the air-sea energy fluxes at the tropical coastal ocean in the southern South China Sea. Data Brief.

[9] Ulses G.A., Smith L.M., Cowles T.J. (2018). The Ocean Observatories Initiative. Oceanography.

[10] Resnick P., Varian H.R. (1997). Recommender systems. Commun. ACM.

[11] Yuan R., Chen G., Li F. (2019). User interest model construction and update for news recommendation. Appl. Res. Comput..

[12] Bao M.Y., Shen J.X. (2019). Design and implementation of music recommendation system based on android. J. Shanxi Datong Univ..

[13] Alshammari G., Kapetanakis S., Alshammari A., Polatidis N., Petridis M. (2019). Improved movie recommendations based on a hybrid feature combination method. Vietnam J. Comput. Sci..

[14] Yu L., Han F., Huang S., Luo Y. (2018). A content-based goods image recommendation system. Multimed. Tools Appl..

[15] Zhang L.M., Liu T.S., Pan S.W., Yang H.Z. (2016). Attribute clustering based collaborative filtering in patient prescription recommendation. Basic Clin. Pharmacol. Toxicol..

[16] Lin W., Alvarez S.A., Ruiz C. (2002). Efficient adaptive-support association rule mining for recommender systems. Data Min. Knowl. Discov..

[17] Kuang L., Yu L., Huang L., Wang Y., Ma P., Li C., Zhu Y. (2018). A Personalized QoS Prediction Approach for CPS Service Recommendation Based on Reputation and Location-Aware Collaborative Filtering. Sensors.

[18] Joanna A.U., Andrea G.O., Ari B.A., Germán M., Miguel G.M., María P.M.O., Antonio J. (2018). HyRA: A hybrid recommendation algorithm focused on smart POI. Ceutí as a study scenario. Sensors.

[19] Ekstrand M.D. (2007). Collaborative Filtering Recommender Systems.

[20] Shu J., Shen X., Liu H., Yi B., Zhang Z. (2017). A content-based recommendation algorithm for learning resources. Multimed. Syst..

[21] Son J., Kim S.B. (2017). Content-based filtering for recommendation systems using multiattribute networks. Expert Syst. Appl..

[22] Hu Z.H., Li X., Wei C., Zhou H.L. (2018). Examining collaborative filtering algorithms for clothing recommendation in e-commerce. Text. Res. J..

[23] Choi I.Y., Oh M.G., Kim J.K., Ryu Y.U. (2016). Collaborative filtering with facial expressions for online video recommendation. Int. J. Inf. Manag..

[24] Bach N.X., Hai N.D., Phuong T.M. (2016). Personalized recommendation of stories for commenting in forum-based social media. Inf. Sci..

[25] Burke R. (2002). Hybrid recommender systems: Survey and experiments. User Model. User Adapt. Interact..

[26] Bozanta A., Kutlu B. (2018). Developing a contextually personalized hybrid recommender system. Mob. Infor. Syst..

[27] Geetha G., Safa M., Fancy C., Saranya D. (2018). A hybrid approach using collaborative filtering and content based filtering for recommender system. J. Phys. Conf..

[28] Fang Q., Xu C., Hossain M.S., Muhammad G. (2016). STCAPLRS: A spatial-temporal context-aware personalized location recommendation system. Acm Trans. Intell. Syst. Technol..

[29] Ceccaroni L., Velickovski F., Blaas M., Wernand M.R., Subirats L. (2018). Artificial intelligence and earth observation to explore water quality in the Wadden Sea. Earth Observation Open Science and Innovation.

[30] Hatzikos E.V., Tsoumakas G., Tzanis G., Bassiliades N., Vlahavas I. (2008). An empirical study on sea water quality prediction. Knowl. Based Syst..

[31] Din E.S.E., Yun Z., Suliman A. (2017). Mapping concentrations of surface water quality parameters using a novel remote sensing and artificial intelligence framework. Int. J. Remote Sens..

[32] Emamgholizadeh S., Kashi H., Marofpoor I., Zalaghi E. (2014). Prediction of water quality parameters of Karoon River (Iran) by artificial intelligence-based models. Int. J. Environ. Sci. Technol..

[33] Han Y., Han Z., Wu J., Yu Y., Gao S., Hua D., Yang A. (2020). Artificial intelligence recommendation system of cancer rehabilitation scheme based on iot technology. IEEE Access.

[34] Zhang L., Luo T., Fei Z., Wu Y. (2018). A recommendation model based on deep neural network. IEEE Access.

[35] Roh T.H., Oh K.J., Han I. (2003). The collaborative filtering recommendation based on SOM cluster-indexing CBR. Expert Syst. Appl..

[36] Ziegler C.-N., Lausen G. (2010). Making product recommendations more diverse. Bull. Tech. Comm. Data Eng..

[37] Deshpande M., Karypis G. (2004). Item-based top-N recommendation algorithms. ACM Trans. Inf. Syst.

[38] Peker S., Kocyigit A. mRHR: A modified reciprocal hit rank metric for ranking evaluation of multiple preferences in top-n recommender systems. Proceedings of the International Conference on Artificial Intelligence: Methodology, Systems, and Applications.

[39] Ziegler C.N., Mcnee S.M., Konstan J.A., Lausen G. Improving Recommendation Lists through Topic Diversification. Proceedings of the International World Wide Web Conference Committee (IW3C2).

[40] Kennish M.J. (2001). Practical Handbook of Marine Science.

[41] Xian T. (2005). The Research of Personalized Recommendation Methods Based on Item Rating Prediction and Classification. Master’s Thesis.

[42] Liu Y., Qiu M., Liu C., Guo Z. Big data in ocean observation: Opportunities and challenges. Proceedings of the 2nd International Conference on Big Data Computing and Communication (BigCom).

[43] Jige G. (2007). Estimation and validation of surface currents in the global ocean from Argo floats. Insititute of Atmospheric Physics.

[44] Small H. (2010). Co-citation in the scientific literature: A new measure of the relationship between two documents. J. Assoc. Inf. Sci. Technol..

[45] Leydesdorff L., Vaughan L. (2006). Co-occurrence matrices and their applications in information science: Extending ACA to the Web environment. J. Am. Soc. Inf. Sci. Technol..

[46] National Marine Data Center. http://mds.nmdis.org.cn/.

[47] China Argo Real-Time Data Center. http://www.argo.org.cn/.

[48] Seafloor Observation Network Experiment System. http://www.dns863.net/oceanview/index.html.

